# Timing of ceiling lift–assisted out-of-bed sitting training and outcomes in critically ill patients: a prospective cohort study

**DOI:** 10.1186/s13054-025-05741-9

**Published:** 2025-11-17

**Authors:** Zhengyong Hu, Yuwen Chen, Liang Xu, Lanqi Guo, Yong Li, Min Mo, Jingyuan Xu, Xiaoqing Li, Yufen Zhu, Xiang Hong, Hongxing Wang, Songqiao Liu

**Affiliations:** 1 Department of Rehabilitation Medicine, Zhongda Hospital, Southeast University, Nanjing, 210009 Jiangsu China; 2 Jiangsu Provincial Key Laboratory of Critical Care Medicine, Department of Critical Care Medicine, Trauma Center, Zhongda Hospital, School of Medicine, Southeast University, Nanjing, 210009 Jiangsu China; 3 The First People’s Hospital of Lianyungang, The Lianyungang Clinical College of Nanjing Medical University, The First Affiliated Hospital of Kangda College of Nanjing Medical University, The Affiliated Lianyungang Hospital of Xuzhou Medical University, Lianyungang, 222000 Jiangsu China; 4 Department of Critical Care Medicine, Trauma Center, Nanjing Lishui People’s Hospital, Zhongda Hospital Lishui Branch, Nanjing, 211200 Jiangsu China; 5 Department of Epidemiology and Health Statistics, School of Public Health, Southeast University, Nanjing, 210009 Jiangsu China

**Keywords:** Ceiling lift, Out-of-bed sitting training, Potential safety events, Mobilization, Critically ill patients

## Abstract

**Background:**

Early mobilization is widely adopted in modern ICU practice, but evidence regarding the safety and clinical outcomes of early ceiling lift-assisted out-of-bed sitting training (OBST) remains limited.

**Methods:**

This prospective observational cohort study was conducted in the ICU of Zhongda Hospital, Nanjing, China. Adults aged 18–80 years who underwent ceiling lift–assisted OBST and had an ICU stay of ≥ 24 h, were categorized by the timing of their first OBST session as early (≤ 72 h after ICU admission) or late (> 72 h after ICU admission). Trained personnel prospectively collected data from electronic health records, session logs, and telephone interviews as appropriate. Propensity score matching was used to adjust for potential confounders. The primary outcome was the rate of potential safety events per OBST session; non-inferiority of early OBST was prespecified with a 2% absolute margin.

**Results:**

Between February 1, 2023, and May 17, 2024, 584 patients were screened, and 396 were enrolled (early: n = 136; late: n = 260). After 1:1 propensity score matching, 125 patients remained in each matched group. Potential safety event rates were 0.36% (early) vs 0.99% (late) per session (risk difference: − 0.63%; 95% CI: − 1.51% to 0.25%). The upper limit of the 95% CI was below the 2% noninferiority margin. Early OBST was associated with greater improvement in FSS-ICU scores (mean difference: 1.31 points, *P* = 0.020), shorter ICU length of stay (mean difference: − 2.75 days, *P* < 0.001), and fewer OBST sessions (mean difference: − 2.02 sessions, *P* < 0.001). Follow-up for 28-day post-ICU mortality was completed in 96.4% of patients.

**Conclusions:**

Early ceiling lift–assisted OBST was associated not with increased safety event rates but with improved functional outcomes and a shorter ICU stay. These findings suggest that early OBST can be used safely in ICU mobilization and warrant evaluation in randomized controlled trials.

**Trial registration:**

Chinese Clinical Trial Registry (ChiCTR), ChiCTR2200066701. Registered on 14 December 2022.

**Supplementary Information:**

The online version contains supplementary material available at 10.1186/s13054-025-05741-9.

## Introduction

Critically ill patients frequently experience delayed initiation of mobilization owing to safety concerns, physiological instability, sedation requirements, and system-level barriers such as staffing shortages and workflow constraints. These factors commonly lead to prolonged bed rest, which is associated with intensive care unit (ICU) delirium, impaired exercise capacity, and ICU-acquired weakness [1–3]. This situation can also reduce lung volume, impair ventilation/perfusion matching, and increase airway resistance, contributing to ventilator-associated and hospital-acquired pneumonia, delayed liberation from mechanical ventilation, and extended ICU length of stay [4].

Early mobilization in critically ill patients has been shown to be safe and associated with shorter hospital stays and improved physical functioning, independence, and quality of life [5–9]. However, this evidence derives primarily from multimodal mobilization programs that bundle in-bed exercises, sitting, standing, and walking, making it difficult to determine the specific contribution of individual components. The transition from supine to upright positioning represents the earliest physiologically meaningful milestone in progressive mobility, associated with improvements in lung volume, end-expiratory lung volume, respiratory compliance, work of breathing, oxygenation, and respiratory muscle strength [10–13]. Out-of-bed sitting training (OBST), defined as a supervised transfer from bed to chair, is the primary clinical maneuver for achieving this initial postural change. Despite its foundational role, the optimal timing for OBST initiation remains undefined, as current recommendations are extrapolated from trials of multimodal mobilization programs rather than studies of OBST alone.

Implementation of OBST is labor-intensive and demands specialized training, particularly for patients with severe neuromuscular weakness. Safety concerns, such as patient falls or provider injuries during assisted transfers, may further delay initiation. Various assistive devices have been used to support early mobilization in ICU settings [14, 15] and have shown clinical benefit [16]. At our center, ceiling lift systems are available throughout the ICU, enabling standardized support for all assisted OBST sessions. Nevertheless, the timing of the first OBST varies across patients due to clinical judgment and workflow factors.

In this prospective observational cohort study, we aimed to compare the safety and clinical outcomes of early versus late ceiling lift–assisted OBST in critically ill patients. We hypothesized that early OBST would be noninferior to late initiation with respect to the rate of potential safety events per OBST session.

## Methods

### Study design and setting

This was a single-center, prospective, observational cohort study conducted in the ICU of Zhongda Hospital, Southeast University, Nanjing, China. Adult patients who underwent ceiling lift–assisted OBST and were eligible for inclusion were enrolled between February 1, 2023, and May 17, 2024. The study was approved by the Institutional Review Board (Approval No. 2022ZDSYLL269-P01). Written informed consent was obtained from all participants or their legally authorized representatives. All procedures were conducted in accordance with the Declaration of Helsinki. The study was prospectively registered at the Chinese Clinical Trial Registry (ChiCTR2200066701). Reporting followed the Strengthening the Reporting of Observational Studies in Epidemiology (STROBE) guidelines.

Patients were categorized according to a pre-specified definition of OBST timing: early (≤ 72 h after ICU admission) or late (> 72 h), as used in two recent meta-analyses [8, 17]. This categorization was based exclusively on the documented time of the first OBST session, which occurred only after (1) the attending intensivist verified predefined physiological eligibility criteria (inclusion criteria 3–4), and (2) the multidisciplinary team confirmed day-specific clinical stability criteria, as detailed in the Procedure section. Reasons for delayed OBST (> 72 h) were systematically documented and are presented in Additional file 1: e-Table 1.

**Table 1:** Clinical and demographic data of the patients

Variables	Before matching				After matching		
	Early OBST N = 136	Late OBST N = 260	*P* value		Early OBST N = 125	Late OBST N = 125	*P* value
Age, median (IQR), years	59.0 (48.0 to 72.0)	65.0 (53.0 to 72.0)	0.040		61.0 (50.0 to 72.0)	63.0 (51.0 to 70.0)	0.944
Male, no. (%)	84 (61.8)	181 (69.6)	0.143		77 (61.6)	77 (61.6)	> 0.999
Body mass index, median (IQR), kg/m^2^	24.2 (22.0 to 26.3)	24.1 (21.7 to 25.9)	0.398		23.7 (21.8 to 26.1)	23.9 (21.1 to 26.1)	0.664
APACHE II score ^a^	12.0 (10.0 to 15.2)	15.0 (12.0 to 20.0)	< 0.001		13.2 ± 4.7	13.0 ± 4.6	0.839
SOFA score, median (IQR)	6.0 (4.0 to 9.0)	7.0 (5.0 to 10.0)	0.004		7.0 (4.0 to 9.0)	6.0 (4.0 to 8.0)	0.956
GCS score, median (IQR)	10.0 (10.0 to 10.0)	10.0 (9.0 to 10.0)	< 0.001		10.0 (10.0 to 10.0)	10.0 (10.0 to 10.0)	0.822
Comorbidities, no. (%)							
Hypertension	68 (50.0)	155 (59.6)	0.084		62 (49.6)	61 (48.8)	> 0.999
Diabetes mellitus	39 (28.7)	78 (30.0)	0.874		36 (28.8)	34 (27.2)	0.888
Stroke	14 (10.3)	49 (18.8)	0.039		14 (11.2)	12 (9.6)	0.836
Coronary heart disease	10 (7.4)	27 (10.4)	0.422		10 (8.0)	12 (9.6)	0.823
Chronic kidney disease	7 (5.1)	31 (11.9)	0.046		7 (5.6)	7 (5.6)	> 0.999
COPD	7 (5.1)	18 (6.9)	0.637		7 (5.6)	7 (5.6)	> 0.999
Primary diagnoses, no. (%)			0.001				0.998
Respiratory system	44 (32.4)	89 (34.2)			42 (33.6)	43 (34.4)	
Circulatory system	30 (22.1)	41 (15.8)			26 (20.8)	27 (21.6)	
Nervous system	12 (8.8)	63 (24.2)			12 (9.6)	12 (9.6)	
Digestive system	22 (16.2)	35 (13.5)			20 (16.0)	18 (14.4)	
Others	28 (20.6)	32 (12.3)			25 (20.0)	25 (20.0)	
Use of vasoactive drugs, no. (%)	27 (19.9)	59 (22.7)	0.601		27 (21.6)	30 (24.0)	0.763
Use of sedative drugs, no. (%)	38 (27.9)	95 (36.5)	0.108		37 (29.6)	43 (34.4)	0.498
Use of CRRT, no. (%)	13 (9.6)	30 (11.5)	0.666		12 (9.6)	14 (11.2)	0.836
Use of invasive ventilation, no. (%)	15 (11.0)	101 (38.8)	< 0.001		15 (12.0)	18 (14.4)	0.709
FSS-ICU score at OBST initiation, median (IQR)	17.0 (8.8 to 21.0)	7.0 (0.0 to 17.0)	< 0.001		16.0 (8.0 to 20.0)	15.0 (7.0 to 20.0)	0.706
Study week of OBST initiation, median (IQR)	33.5 (19.0 to 47.2)	34.0 (15.0, 48.2)	0.473		34.0 (19.0 to 48.0)	36.0 (18.0 to 49.0)	0.751
Timing of OBST initiation, median (IQR), d	2.0 (1.0 to 3.0)	8.0 (6.0 to 11.0)	< 0.001		2.0 (1.0 to 3.0)	7.0 (5.0 to 9.0)	< 0.001

APACHE II = Acute Physiology and Chronic Health Evaluation II; COPD = chronic obstructive pulmonary disease; CRRT = continuous renal replacement therapy; FSS-ICU = Functional Status Score for the Intensive Care Unit; GCS = Glasgow Coma Scale; ICU = intensive care unit; IQR = interquartile range; OBST = out-of-bed sitting training; SD = standard deviation; SOFA = Sequential Organ Failure Assessment

^a^Values for APACHE II score are expressed as median (IQR) or mean (SD), as appropriate

No missing data for any variable

### Patients

The inclusion criteria were as follows: (1) aged between 18 and 80 years; (2) admitted to the ICU for at least 24 h; (3) fulfilled the criteria for early mobilization initiation (temperature < 38.5 °C, heart rate 60–120 beats per minute, systolic blood pressure 90–180 mmHg, diastolic blood pressure ≤ 110 mmHg, mean arterial pressure ≥ 65 mmHg, positive end-expiratory pressure ≤ 10 cmH_2_O, fraction of inspired oxygen [FiO_2_] < 60%, respiratory rate ≤ 35 breaths per minute, peripheral blood oxygen saturation [SpO_2_] ≥ 90%, and no or low-dose vasoactive drugs [epinephrine/norepinephrine < 0.1 µg/kg/min, and dopamine < 5 µg/kg/min]) [18, 19]; (4) deemed clinically appropriate for OBST by the attending intensivists (a protocol-guided determination that criterion (3) was satisfied, that no guideline-recommended contraindications to OBST were present, and that the patient’s treatment goals were aligned with recovery and rehabilitation rather than comfort care alone) [18, 19]; and (5) provided informed consent.

We excluded patients with any of the following conditions: (1) body mass index (BMI) ≥ 40 kg/m^2^; (2) ICU readmission; (3) invasive ventilation lasting longer than 24 h before ICU admission (to minimize potential misclassification of OBST timing as early versus late); (4) severe cardiac dysfunction (left ventricular ejection fraction < 20%); (5) agitation preventing cooperation; (6) presence of life-support devices incompatible with OBST (e.g., extracorporeal membrane oxygenation, intra-aortic balloon pump, etc.); (7) contraindications to undergoing OBST (e.g., new-onset spinal injury, unstable pelvic or lower extremity fractures, etc.); or (8) pregnancy.

### Procedure

The ceiling lift system (Guldmann, Denmark) was incorporated into routine ICU care in July 2022. Prior to clinical implementation, all nursing and physiotherapy staff completed a standardized, hands-on training program delivered by ICU clinical educators, rehabilitation specialists and manufacturer-certified technical specialists, focused on safe patient handling, mobilization techniques, and proper operation of the ceiling lift system. The training included supervised practice, and only personnel who demonstrated safe and competent performance were permitted to perform OBST. Attending intensivists received formal training on the institutional protocol for OBST and applied its eligibility criteria consistently during daily assessments. By the time data collection began on February 1, 2023, the multidisciplinary team had accrued more than 7 months of operational experience with the system.

For patients deemed eligible for OBST, the decision to proceed with each daily session was based on multidisciplinary assessment: the attending intensivist confirmed the absence of new medical contraindications, while nursing and physiotherapy staff evaluated real-time physiological tolerance against predefined safety thresholds (e-Table 2). During sessions, patients were transferred from supine to a seated position using the ceiling lift. The intensivist monitored vital signs continuously, while nursing and physiotherapy personnel executed the transfer following a detailed institutional protocol specifying sling selection, patient positioning, management of lines and tubes, and team coordination (Additional file 1).

**Table 2:** Primary outcome and secondary outcomes after propensity score matching

Variables	Difference OR Odds Ratio (reference: Late OBST)	Standard Error	95% CI	*P* value
**Primary outcome**				
Potential safety events rate, %	RD: − 0.631	0.005	− 1.514 to 0.252	** < 0.001**
**Secondary outcomes**				
Change in FSS-ICU score	MD: 1.312	0.560	0.209 to 2.415	**0.020**
Change in arterial blood gas				
PaO_2,_ mmHg	MD: − 2.390	3.839	− 9.951 to 5.172	0.534
PaCO_2,_ mmHg	MD: 0.422	0.850	− 1.260 to 2.105	0.620
PaO_2_/FiO_2_, mmHg	MD: − 7.737	11.106	− 29.609 to 14.136	0.487
Lac, mmol/L	MD: 0.005	0.116	− 0.223 to 0.233	0.967
Duration per OBST session, min	MD: 3.644	2.331	− 0.929 to 8.218	0.118
Number of OBST sessions	MD: − 2.024	0.556	− 3.118 to − 0.930	** < 0.001**
ICU length of stay, d	MD: − 2.752	0.691	− 4.119 to − 1.385	** < 0.001**
Ventilator-free days	MD: 6.000	4.445	−4.510 to 16.510	0.219
Weaning success	OR: 1.000	1.414	0.063 to 15.988	> 0.999
ICU mortality ^a^	—	—	—	0.500
28 − day post-ICU mortality ^b^	OR: 0.333	0.816	0.067 to 1.652	0.178

FiO_2_ = fraction of inspired oxygen; FSS-ICU = Functional Status Score for the Intensive Care Unit; ICU = intensive care unit; IQR = interquartile range; Lac = lactate; MD = mean difference; OBST = out-of-bed sitting training; OR = Odds Ratio; PaCO_2_ = arterial carbon dioxide partial pressure; PaO_2_ = arterial oxygen partial pressure; RD = risk difference

^a^*P*-value based on exact McNemar test

^b^Data on 28-day post-ICU mortality were missing for 4 patients with early OBST and 5 with late OBST

Bold values indicate statistically significant differences between Early OBST and Late OBST groups (p < 0.05)

Each OBST session was terminated if one of the following occurred: (1) clinical signs of intolerance developed during the session (e-Table 3), or (2) the session reached a target duration of 30–60 min. If physiological parameters remained stable and the patient tolerated further sitting, sessions could extend beyond 60 min. After the session ended, patients were returned to the supine position and monitored for 30 min.

In accordance with institutional protocol, patients underwent 1–2 OBST sessions daily when clinically indicated, and OBST was discontinued upon ICU discharge, irreversible clinical deterioration, or 28 days after enrollment, whichever came first. The frequency of repeat sessions was determined daily through clinical reassessment, guided by the absence of new-onset contraindications to OBST.

### Data collection

All data were prospectively recorded by independent, trained personnel who were unaware of patients’ categorization based on OBST timing. At enrollment, baseline demographics and clinical variables were extracted from the electronic health record, including age, sex, BMI, Acute Physiology and Chronic Health Evaluation II (APACHE II) score, Sequential Organ Failure Assessment (SOFA) score, and a modified Glasgow Coma Scale (GCS) based solely on eye-opening and motor response components (range 2–10), as the presence of an artificial airway precluded reliable verbal assessment. Additional variables included comorbidities, primary diagnosis, and use of vasoactive agents, sedatives, continuous renal replacement therapy, or invasive ventilation.

During each OBST session, potential safety events and session-specific parameters were documented in real time by trained research observers who were not involved in clinical care or decision-making. Baseline Functional Status Score for the Intensive Care Unit (FSS-ICU) and arterial blood gas parameters were recorded at enrollment, and follow-up measurements were obtained after the last OBST session. ICU-related outcomes, including ICU length of stay, duration of invasive ventilation, weaning outcome, and ICU mortality, were extracted from hospital electronic health records. For 28-day post-ICU mortality, data were ascertained from hospital records or telephone interviews, as appropriate.

### Outcomes

The primary outcome was the rate of potential safety events per OBST session. A potential safety event was defined as a clinical deterioration or any physiological parameter exceeding pre-established safety thresholds [20]. Serious adverse events were defined as death, life-threatening conditions, persistent or substantial disability or incapacity, or hospitalization or prolongation of existing hospitalization.

Potential safety events occurring during OBST sessions were classified according to previously established criteria [20], including the following categories: (1) hemodynamic events (heart rate variation exceeding 20% from baseline, new-onset cardiac arrhythmias, or mean arterial pressure > 140 mmHg or < 55 mmHg); (2) respiratory events (peripheral oxygen saturation [SpO₂] < 88%, sustained SpO₂ decline > 4% lasting ≥ 3 min, or pneumothorax); (3) neurological events (loss of consciousness); (4) removal events (feeding tubes; ventricular, thoracic, abdominal, or limb drains; arterial or venous catheters; or artificial airways); and (5) other events (dizziness or vertigo, falls, or cardiopulmonary arrest). The event rate was calculated as the number of potential safety events divided by the total number of OBST sessions performed.

Secondary outcomes included changes in physical function (measured using the Functional Status Score for the Intensive Care Unit [FSS-ICU]) and arterial blood gas parameters, both defined as the difference between baseline values and those measured after the last OBST session. Additional secondary outcomes included duration per OBST session, number of OBST sessions, ICU length of stay, ventilator-free days, weaning success, ICU mortality, and 28-day post-ICU mortality. All outcome definitions were prespecified and detailed in e-Table 4.

### Statistical analysis

Sample size calculation was based on a prespecified noninferiority hypothesis for the primary safety outcome. We assumed a 1.77% rate of potential safety events per OBST session in late OBST, derived from historical data in prior studies [2, 21], and set a clinically acceptable noninferiority margin of 2.0%. Using PASS software, with a one-sided α of 0.025 and 80% power, we estimated that a total of 683 OBST sessions would be required. Assuming a median of six sessions per patient, this translated to 114 patients with early OBST and 114 with late OBST. To account for anticipated attrition and ensure adequate statistical power after propensity score matching (PSM), we inflated the target sample size by 15%, resulting in a final target of 131 patients with early OBST and 131 with late OBST.

To minimize confounding, we performed 1:1 nearest-neighbor propensity score matching without replacement, using a caliper width of 0.2 standard deviations of the logit of the propensity score. Propensity scores were estimated via logistic regression incorporating the following covariates: age, sex, BMI, APACHE II score, and SOFA score (both calculated within 24 hours of ICU admission); comorbidities; primary diagnosis; as well as GCS score and use of vasopressors, sedatives, continuous renal replacement therapy, invasive ventilation, and baseline FSS-ICU score, all assessed prior to the first out-of-bed sitting session. Covariate balance after matching was assessed using standardized mean differences, with values < 0.1 considered indicative of adequate balance.

Normality of continuous variables was evaluated using the Shapiro–Wilk test. The primary outcome was analyzed for noninferiority using the risk difference. In the unmatched cohort, the two-sided 95% confidence interval (CI) was calculated using the Miettinen-Nurminen method. In the matched cohort, the CI was derived from a binomial regression model with identity link and cluster-robust standard errors (clustered by matched pair). Noninferiority was declared if the upper limit of the 95% CI fell below the prespecified noninferiority margin of 2.0%. As a sensitivity analysis, the risk difference in the matched cohort was re-estimated using the same model adjusted for study week.

For secondary outcomes, continuous variables were analyzed using linear regression in the unmatched cohort and linear mixed-effects models with a random intercept for matched pair; binary outcomes were compared using Fisher’s exact test in the unmatched cohort and conditional logistic regression stratified by matched pair in the matched cohort, with exact McNemar test used when no events occurred in one group. Sensitivity analyses adjusted all matched models for study week (as a continuous variable), except when insufficient events precluded stable estimation.

To evaluate the robustness of results to potential residual confounding, the association between OBST timing (early vs. late) and change in FSS-ICU score was assessed using a multivariable linear mixed-effects model, adjusted for six prespecified covariates: age, GCS score, APACHE II score, sedative use, invasive ventilation, and baseline FSS-ICU score at OBST initiation. Results are reported as adjusted mean differences with 95% CIs. Effect modification was evaluated by testing interaction terms between OBST timing and each covariate.

Clinically relevant binary endpoints, including an improvement of ≥ 5 points in FSS-ICU score from baseline to the assessment after the last OBST session [22], and achieving an FSS-ICU score ≥ 19 after the last OBST session [23], were analyzed using the same conditional logistic regression framework. Results are presented as odds ratios with 95% CIs.

All analyses were conducted in R version 4.4.2 (http://www.R-project.org). Patients lost to follow-up for 28-day post-ICU discharge mortality were excluded from the mortality analysis. No imputation was performed for missing data. Two-sided P values < 0.05 were considered statistically significant. For each patient, study week was defined as the 7-day interval containing the date of first OBST session, counted from enrollment start (February 1, 2023), such that week 1 included days 1–7, week 2 days 8–14, week 3 days 15–21. For patients who died within 28 days of enrollment, ventilator-free days and ICU length of stay were assigned values of 0 and 28 days, respectively.

## Results

### Study population

**Fig. 1:**
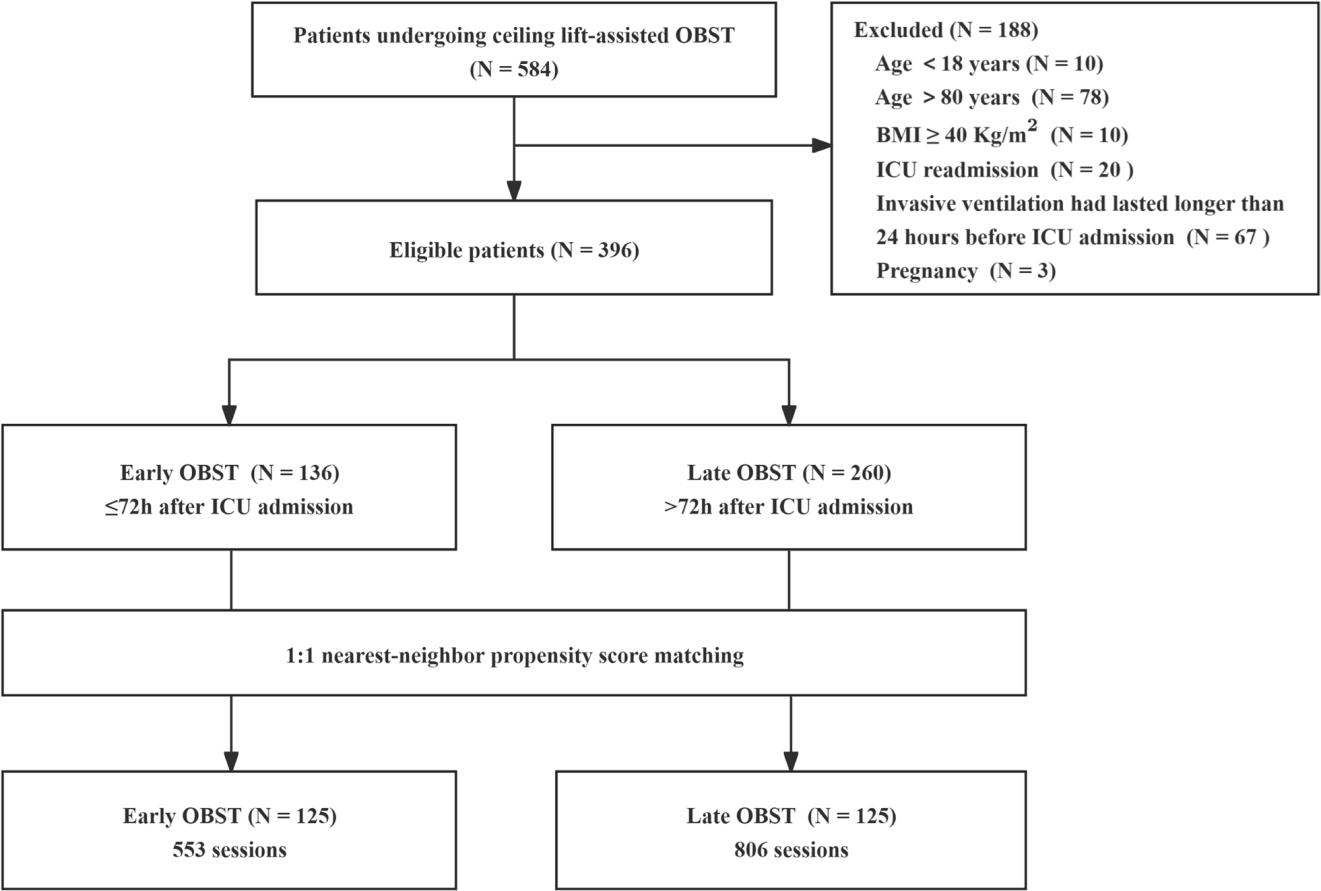
Patient flow through the study BMI = body mass index; ICU = intensive care unit; OBST = out-of-bed sitting training

Between February 1, 2023, and May 17, 2024, 584 patients were screened for eligibility. Of these, 188 patients were excluded according to predefined criteria: age < 18 years (n = 10), age > 80 years (n = 78), BMI ≥ 40 kg/m^2^ (n = 10), ICU readmission (n = 20), prior invasive ventilation lasting > 24 h before ICU admission (n = 67), or pregnancy (n = 3). A total of 396 patients were included in the final analysis. After 1:1 propensity score matching, 125 patients with early OBST were matched to 125 with late OBST. Patients with early OBST underwent a total of 553 OBST sessions, compared with 806 sessions among those with late OBST (Fig. 1). Among the 248 patients who survived to ICU discharge, follow-up for 28-day post-ICU mortality was complete for 239 patients (96.4%), with a maximum follow-up duration of 28 days. Mortality data were missing for four patients with early OBST and five with late OBST due to loss to post-discharge tracking.

Before matching, patients with early OBST were younger, had lower disease severity (as measured by APACHE II and SOFA scores), higher GCS scores (motor and eye components), lower rates of invasive ventilation use, and better baseline physical function compared with those with late OBST. After propensity score matching, baseline characteristics were well balanced between the two matched groups (Table 1; e-Table 5). The median age was 61.0 years (IQR, 50.2 to 71.0), and 61.6% were male. Median APACHE II and GCS scores were 13.0 (IQR, 10.0 to 16.0) and 10.0 (IQR, 10.0 to 10.0), respectively. At OBST initiation, the median FSS-ICU score was 16.0 (IQR, 8.0 to 20.0), and 13.2% of patients were receiving invasive ventilation. As expected, patients with early OBST underwent their first session significantly earlier than those with late OBST (median [IQR]: 2.0 [1.0–3.0] vs. 7.0 [5.0–9.0] days; P < 0.001). The post-matching distribution of GCS scores is presented in e- Figure 1.

### Primary outcome

Before propensity score matching, 15 potential safety events occurred across 2,578 OBST sessions in 396 patients, including 12 hemodynamic and 3 respiratory events. The overall rate was 0.58% (0.51% among patients with early OBST and 0.60% among those with late OBST). The risk difference between early and late OBST was − 0.10% (95% CI: − 0.76% to 0.57%) (e-Tables 6 and 7).

In the matched cohort (250 patients, 1,359 sessions), 10 potential safety events occurred: 8 hemodynamic and 2 respiratory events. The overall rate was 0.74% (0.36% among patients with early OBST and 0.99% among those with late OBST) (e-Table 6). The risk difference between patients with early and late OBST was − 0.63% (95% CI: − 1.51% to 0.25%). The upper limit of the 95% CI fell below the prespecified noninferiority margin of 2.0% (Table 2). A sensitivity analysis adjusting for study week showed consistent results (e-Table 8).

No serious adverse events were identified. All recorded physiological perturbations resolved without intervention following session termination, and no instances of cardiac arrhythmia, mean arterial pressure > 140 mm Hg, pneumothorax, unplanned device dislodgement, or other protocol-defined safety criteria occurred.

### Secondary outcomes

Before propensity score matching, unadjusted comparisons revealed that patients with early OBST had greater improvements in FSS-ICU scores, longer duration per OBST session, fewer total OBST sessions, more ventilator-free days at day 28, shorter ICU length of stay, and lower 28-day post-ICU mortality (e-Tables 7 and 9).

After matching, patients with early OBST demonstrated greater improvement in FSS-ICU score (mean difference: 1.31 points; 95% CI: 0.21 to 2.42; *P* = 0.020; median: 6.0 vs. 4.0 points), required fewer total OBST sessions (mean difference: − 2.02; 95% CI: − 3.12 to − 0.93; *P* < 0.001; median: 4.0 vs. 5.0 sessions), and had shorter ICU length of stay (mean difference: − 2.75 days; 95% CI: − 4.12 to − 1.39; *P* < 0.001; median: 4.0 vs. 6.0 days) (Table 2; e-Table 9). Sensitivity analyses adjusting for study week showed consistent results for these secondary outcomes (e-Table 8).

**Fig. 2:**
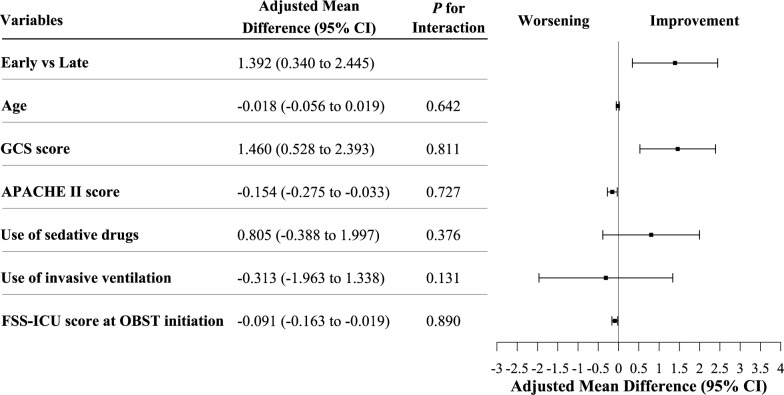
Main and interaction effects of early versus late OBST on FSS-ICU change APACHE II = Acute Physiology and Chronic Health Evaluation II; CI = confidence interval; FSS-ICU = Functional Status Score for the Intensive Care Unit; GCS = Glasgow Coma Scale; OBST = out-of-bed sitting training

In sensitivity analyses to assess robustness to residual confounding, after adjustment for age, GCS score, APACHE II score, sedative exposure, invasive ventilation, and baseline FSS-ICU score, early OBST remained associated with greater FSS-ICU improvement (adjusted mean difference: 1.39 points; 95% CI: 0.34 to 2.45). Higher GCS score was independently associated with greater FSS-ICU improvement (adjusted mean difference: 1.46 points per point; 95% CI: 0.53 to 2.39), whereas higher APACHE II score (adjusted mean difference: − 0.15 points per point; 95% CI: − 0.28 to − 0.03) and higher baseline FSS-ICU score (adjusted mean difference: − 0.09 points per point; 95% CI: − 0.16 to − 0.02) were associated with less improvement (Fig. 2). No significant interaction was found between OBST timing and any prespecified covariate (all *P*-interaction > 0.05).

Patients with early OBST had significantly higher odds of achieving a clinically meaningful improvement of ≥ 5 points in FSS-ICU score compared with those with late OBST (odds ratio [OR]: 1.95; 95% CI: 1.15 to 3.30; *P* = 0.013). There was no significant difference in the odds of achieving an FSS-ICU score of ≥ 19 points after the last OBST session between patients with early and late OBST (OR: 1.44; 95% CI: 0.79 to 2.63; *P* = 0.230) (e-Figure 2).

No significant differences were observed between patients with early and late OBST in the duration per OBST session, arterial blood gas parameters, ventilator-free days, weaning success, ICU mortality, or 28-day post-ICU mortality (Table 2). Changes in FSS-ICU domain scores and arterial blood gas parameters are presented in e-Figs. 3–6, respectively.

## Discussion

In this prospective observational cohort study, among patients who underwent ceiling lift-assisted OBST, the rate of potential safety events per session was low, and no serious adverse events occurred. In the propensity score–matched analysis, early OBST was not associated with an increased risk of these events compared with late OBST. Patients with early OBST also had greater improvement in FSS-ICU scores and shorter ICU length of stay.

The low rate of potential safety events likely reflects OBST occurring within a structured, intensivist-led mobilization program rather than in isolation. Transient heart rate fluctuations and mild oxygen desaturation were infrequent, self-resolving, and consistent with previous reports of early mobilization [5, 20]. Importantly, all OBST sessions were based on bedside physiological assessments rather than a fixed schedule, unlike rigid trial protocols linked to higher adverse events or mortality [2, 24, 25]. While recent data suggest that out-of-bed activity can be safely initiated at norepinephrine doses ≤ 0.20 µg/kg/min [26], patients requiring higher levels of vasopressor support were not included in this cohort, which may have contributed to observed hemodynamic stability. The use of ceiling lifts may have further reduced the metabolic cost of transfer [27], lessening both staff exertion and patient stress, factors that may help explain the observed safety during OBST.

Early initiation of out-of-bed sitting, with a median start at 2 days after ICU admission, was associated with greater improvement in FSS-ICU scores, despite a lower total number of sessions among patients with early OBST. This timing falls within the 24- to 72-h window that has been consistently linked to more favorable functional trajectories in critically ill patients [6, 7, 28, 29]. These findings suggest that achieving the first out-of-bed milestone early, rather than the total number of OBST sessions, may be more important for early functional recovery [9, 30]. Additionally, patients with higher baseline FSS-ICU scores showed smaller improvements in multivariable analysis, consistent with a ceiling effect in functional recovery.

In multivariable analysis, early OBST remained associated with greater functional improvement after adjustment for prespecified clinical covariates. Higher GCS at initiation was independently associated with greater FSS‑ICU improvement, likely reflecting greater baseline neurological integrity. Importantly, the association between earlier initiation and functional improvement did not differ across levels of consciousness (interaction *P* > 0.05), aligning with a randomized trial showing improved functional independence with early mobilization even in patients with GCS ≤ 8 [31]. Although higher APACHE II scores were associated with attenuated functional gains, as expected in more severely ill patients [6, 7, 29], the association between early OBST and greater functional improvement persisted after adjustment and showed no evidence of effect modification by illness severity (interaction *P* > 0.05).

Patients with early OBST were more likely to achieve a clinically meaningful improvement of ≥ 5 points in the FSS-ICU score, exceeding the established minimal important difference [22]. In contrast, the odds of reaching an FSS-ICU score of ≥ 19 after the last OBST session did not differ between early OBST and late OBST, even though this threshold has been associated with a greater likelihood of discharge home in a prior study [23]. This lack of difference may reflect the characteristics of our study population. In middle-aged, mixed-etiologic ICU cohorts, prior evidence suggests that final functional status is more strongly associated with whether standing or ambulation occurred during the ICU stay than with the timing of the first OBST session alone [7].

Early OBST was associated with shorter ICU length of stay and greater improvement in FSS-ICU scores in our cohort. Although the FSS-ICU was not used to guide discharge decisions in our unit, this dual association is consistent with mounting evidence that early mobilization is associated with better functional status (as measured by the FSS-ICU or similar tools) and reduced ICU length of stay [8, 9, 29, 32]. Ceiling lifts facilitated OBST in our high-resource setting, but the procedure does not require specialized equipment and can be safely performed using sliding boards [27]. Together, these results indicate that early, physiology-guided OBST, as observed in our cohort, may represent a clinically meaningful initial functional milestone that could be considered in various ICU settings.

This study has several limitations. First, as a prospective observational cohort study, the observed associations may be affected by unmeasured or residual confounding, despite the use of propensity score matching to balance observed confounders. Nevertheless, the prospective design in a real-world ICU setting may enhance the clinical relevance of our findings. Second, during the study period, the ICU routinely used a ceiling lift-assisted OBST approach that included standardized procedures for transfer, positioning, monitoring, and termination criteria. However, the specific activities performed while sitting (e.g., spontaneous limb movements, engagement with staff), duration of each session, and total number of sessions were not fixed by protocol but instead varied according to individual patient condition and ICU length of stay. This variability may limit the reproducibility of the observed associations. Third, the single-center design may restrict the generalizability of our findings to other settings with different practice patterns or patient demographics. Future multicenter studies in diverse populations are needed to confirm these findings.

## Conclusions

In this prospective observational cohort study, early ceiling lift-assisted OBST was not associated with an increased rate of potential safety events compared with late OBST. It was associated with greater improvement in FSS-ICU scores and shorter ICU length of stay.

## Supplementary Information

Additional file 1.

Additional file 2.

## Data Availability

The datasets used and analyzed during the current study are not publicly available owing to ongoing studies but are available from the corresponding author upon reasonable request.

## Abbreviations

APACHE II: Acute physiology and chronic health evaluation II
CI: Confidence interval
FSS-ICU: Functional status score for the intensive care unit
GCS: Glasgow coma scale
ICU: Intensive care unit
IQR: Interquartile range
OBST: Out-of-bed sitting training
PSM: Propensity score matching
