# Supplementary material for: Timing of ceiling lift–assisted out-of-bed sitting training and outcomes in critically ill patients: a prospective cohort study
Source: Crit Care. 2025 Nov 17;29:493. doi: 10.1186/s13054-025-05741-9 (PMC12625302; doi:10.1186/s13054-025-05741-9)
Supplement: Supplementary file 1 — Additional file 1. [file 13054_2025_5741_MOESM1_ESM.docx]

**Timing of Ceiling Lift–Assisted Out-of-Bed Sitting Training and Outcomes in Critically Ill Patients: A Prospective Cohort Study**

Zhengyong Hu^1^, Yuwen Chen^1^, Liang Xu^1^, Lanqi Guo^2^, Yong Li^3^, Min Mo^2,4^, Jingyuan Xu^2^, Xiaoqing Li^2^, Yufen Zhu^2^, Xiang Hong^5^, Hongxing Wang^1^, Songqiao Liu^2,3,4*^

***CORRESPONDENCE TO:**

Songqiao Liu, email: [liusongqiao@ymail.com](mailto:liusongqiao@ymail.com) and [101012487@seu.edu.cn](mailto:101012487@seu.edu.cn)

[Institutional protocol for ceiling lift-assisted OBST 1](#_Toc210838492)

[e-Table 1. Reasons for delayed OBST 3](#_Toc210838493)

[e-Table 2. Daily safety screening checklist for ceiling lift–assisted OBST 4](#_Toc210838494)

[e-Table 3. Termination criteria for ceiling lift-assisted OBST 5](#_Toc210838495)

[e-Table 4. List of trial outcomes and definitions 6](#_Toc210838496)

[e-Table 5. Characteristics of patients with early versus late OBST before and after matching 8](#_Toc210838497)

[e-Table 6. Types and incidence of potential safety events 10](#_Toc210838498)

[e-Table 7. Primary outcome and secondary outcomes before propensity score matching 11](#_Toc210838499)

[e-Table 8. Sensitivity analysis of outcomes by study week in the propensity score–matched cohort 12](#_Toc210838500)

[e-Table 9. Secondary outcomes 13](#_Toc210838501)

[e-Figure 1. GCS score distribution according to OBST timing 14](#_Toc210838502)

[e-Figure 2. Odds ratios for functional outcomes according to OBST timing 15](#_Toc210838503)

[e-Figure 3. Changes in FSS-ICU domain scores among patients with early OBST 16](#_Toc210838504)

[e-Figure 4. Changes in FSS-ICU domain scores among patients with late OBST 17](#_Toc210838505)

[e-Figure 5. Changes in arterial blood gas parameters among patients with early OBST 18](#_Toc210838506)

[e-Figure 6. Changes in arterial blood gas parameters among patients with late OBST 19](#_Toc210838507)

[References: 20](#_Toc210838508)

## Institutional protocol for ceiling lift-assisted OBST

At the start of each session, patients were placed supine, with the physiotherapist and nurse standing at each side of the bed. A basic high-back sling was laid flat beneath the patient by rolling the patient from side to side, with the upper edge aligned at the occipital protuberance. Based on patient size, sling straps of appropriate length were attached to the lifting hanger, with the lower straps crossed to reduce the risk of slippage.

Indwelling lines, tubes, and drains were arranged by nursing staff to ensure safety during transfer. The sling was then lifted to transition the patient from supine to seated, followed by transfer to a bedside wheelchair. Seated posture was adjusted to maintain neutral trunk alignment and feet flat on the floor. A seatbelt was used in most sessions for additional support. Throughout OBST, the intensivist monitored vital signs, the nurse managed lines, and the physiotherapist oversaw positioning.

In the event of a potential safety event, the clinical team responded with interventions including supine repositioning, supplemental oxygen, airway suctioning, maintenance of airway patency, wound sealing, tube management, cardiopulmonary resuscitation, defibrillation, venous access placement, and stabilization of injured regions, all of which were aimed at maintaining hemodynamic and respiratory stability. All such events and interventions were documented in real time within the electronic medical record as part of standard clinical care.

For conscious patients, anxiety-reduction strategies were employed as needed, including verbal reassurance, guided breathing, environmental adjustments (e.g., noise reduction, lighting control), therapeutic positioning for comfort, focused communication, and continuous emotional support from nursing or caregiving staff.

All procedures, responses, and clinical decisions were documented in the medical record as part of standard care and later abstracted for study purposes.

## e-Table 1. Reasons for delayed OBST

|  | **Before matching, no. (%) N = 260** | **After matching, no. (%)**  **N = 125** |
| --- | --- | --- |
| **Patient Factors** |  |  |
| Physiological instability | 81 (31.2) | 35 (28.0) |
| Fatigue | 20 (7.7) | 5 (4.0) |
| Sacral skin pressure ulcer | 5 (1.9) | 1 (0.8) |
| Deep vein thrombosis | 4 (1.5) | 4 (3.2) |
| Delirium/agitation | 10 (3.8) | 8 (6.4) |
| Patient refusal/anxiety | 8 (3.1) | 7 (5.6) |
| Medical procedures/orders | 15 (5.8) | 10 (8.0) |
| Surgical contraindication | 8 (3.1) | 2 (1.6) |
| **Intensive Care Team Factors** |  |  |
| Anticipated risks | 71 (27.3) | 36 (28.8) |
| Femoral lines | 8 (3.1) | 4 (3.2) |
| **Organizational Factors** |  |  |
| Time constraints | 15 (5.8) | 7 (5.6) |
| Limitations related to the device | 15 (5.8) | 6 (4.8) |

**Abbreviation:** OBST= out-of-bed sitting training.

## e-Table 2. Daily safety screening checklist for ceiling lift–assisted OBST

| **Indications** | **Contraindications** |
| --- | --- |
| Respiratory rate ≤ 35 breaths per minute | Prone positioning |
| SpO_2_ ≥ 90% | Pacemaker-dependent rhythm (transvenous or epicardial) |
| FiO_2_ <0.6 | Bradycardia requiring pharmacologic support or emergent pacing |
| Positive end-expiratory pressure ≤10 cmH_2_O | Femoral intra-aortic balloon pump |
| Ventricular rate 60 to120 beats per minute | Femoral extracorporeal membrane oxygenation |
| Epinephrine/norepinephrine <0.1 µg/kg/min | Cardiac ischemia (ongoing chest pain/ECG changes) |
| Dopamine <5 µg/kg/min | IV antihypertensive therapy for hypertensive emergency |
| Systolic blood pressure 90 to 180 mmHg | Left ventricular ejection fraction <20% |
| Diastolic blood pressure ≤110 mmHg | ICP not within target range |
| Mean arterial pressure ≥65 mmHg | Deep sedation (RASS <–2) |
| Temperature <38.5 °C | Agitation preventing cooperation (RASS ＞+2) |
|  | New-onset spinal injury |
|  | Unstable pelvic or lower extremity fractures |
|  | Lumbar drain open (not clamped) |
|  | Uncontrolled seizures |
|  | Large open surgical wound |
|  | Known uncontrolled active bleeding |
|  | Femoral sheath |

**Abbreviations:** ECG = electrocardiogram; FiO_2_ = fraction of inspired oxygen; ICP = intracranial pressure; IV = intravenous; OBST= out-of-bed sitting training; RASS = Richmond Agitation-Sedation Scale; SpO_2_ = peripheral blood oxygen saturation.
Criteria were derived from international consensus[1] and updated clinical guidelines[2].

## e-Table 3. Termination criteria for ceiling lift-assisted OBST

| **Category** | **Event Definition** |
| --- | --- |
| Subjective symptoms | - Severe or intolerable pain requiring analgesic intervention - Patient-reported dyspnea (e.g., on Borg scale ≥3) - Intolerable fatigue (patient unable to sustain upright posture) |
| Neurological system | - Reduced consciousness level compared to the start - RASS ≤− 3 or RASS ＞+2 |
| Respiratory system | - Respiratory rate <5 breaths/min or >40 breaths/min - SpO_2_ <88% - Patient-ventilator dyssynchrony - Risk of accidental or unplanned extubation of the endotracheal tube |
| Cardiovascular system | - Heart rate increase ≥20% from baseline or >130 bpm - Systolic blood pressure <90 mmHg or >180 mmHg - Mean arterial pressure <60 mmHg or >100 mmHg - New-onset arrhythmia requiring treatment - Suspected myocardial ischemia |
| Device | - Risk of catheter removal (or actual removal) - Active bleeding at catheter insertion site requiring intervention |
| Other | - Patient refusal or request to stop mobilization - Active bleeding - Wound dehiscence |

**Abbreviations:** OBST= out-of-bed sitting training; RASS = Richmond Agitation-Sedation Scale; SpO_2_ = peripheral blood oxygen saturation.

Termination criteria were derived from the Japanese Clinical Practice Guidelines for Rehabilitation in Critically Ill Patients 2023 (J-ReCIP 2023)[2]

##

## e-Table 4. List of trial outcomes and definitions

| **Data** | **Definition** | |
| --- | --- | --- |
| **Baseline**  **Characteristics** | Included demographics, severity scores (APACHE II, SOFA, GCS), comorbidities, primary diagnoses, organ support, physical function at OBST initiation, and study week of OBST initiation. | |
| **Primary**  **outcome** | Potential safety events | Clinical deterioration or any predefined event exceeding prespecified safety thresholds, including hemodynamic events, respiratory events, neurological events, device removal-related events, and other adverse occurrences. |
| **Secondary**  **outcomes** | Physical function | Assessed using the FSS-ICU, which evaluates five mobility tasks: rolling, transfer from supine to sit, sitting at the edge of the bed, transfer from sit to stand, and walking. Each item is scored on an 8-point ordinal scale (0 = not able to perform, 7 = complete independence), yielding a total score between 0 and 35, with higher scores reflecting better physical function. Changes in FSS-ICU score were defined as the difference between values at OBST initiation and those at protocol completion. |
|  | Arterial blood gas parameters | Included PaO₂, PaCO₂, PaO₂/FiO₂, and Lac. Changes in arterial blood gas parameters were defined as the difference between values at OBST initiation and those at protocol completion. |
|  | Duration per OBST session (minutes) | Time from first trunk lift-off from bed to full supine return to bed. |
|  | Number of OBST sessions | Total number of OBST sessions received per patient from study enrollment until ICU discharge or study day 28, whichever came first. |
|  | Ventilator-free days | The total number of days that patient is alive and free of ventilation between study enrollment and day 28. Patients who died within 28 days of study enrollment were assigned 0 days. |
|  | ICU length of stay (days) | Defined as the time from study enrollment to ICU discharge or day 28, whichever occurred first, among patients who survived. For patients who died within 28 days of enrollment, ICU length of stay was defined as 28 days. |
|  | Weaning success | Defined as successful discontinuation of mechanical ventilation from study enrollment to ICU discharge or day 28. Intubated: 7-day no reintubation or death, regardless of NIV; or ICU discharge without invasive mechanical ventilation, whichever comes first. Tracheostomized: 7-day spontaneous breathing or ICU discharge with spontaneous breathing, whichever comes first. |

**Abbreviations:** APACHE II = Acute Physiology and Chronic Health Evaluation II; BMI = body mass index; COPD = chronic obstructive pulmonary disease; CRRT = continuous renal replacement therapy; FiO_2_ = fraction of inspired oxygen; FSS-ICU = Functional Status Score for the Intensive Care Unit; GCS = Glasgow Coma Scale; ICU = intensive care unit; OBST= out-of-bed sitting training; PaCO_2_ = arterial carbon dioxide partial pressure; PaO_2_ = arterial oxygen partial pressure; SOFA = Sequential Organ Failure Assessment.

## e-Table 5. Characteristics of patients with early versus late OBST before and after matching

| **Variables** | **Before matching** | | |  | **After matching** | | |
| --- | --- | --- | --- | --- | --- | --- | --- |
|  | **Early OBST** | **Late OBST** | **SMD** |  | **Early OBST** | **Late OBST** | **SMD** |
| **Number** | 136 | 260 |  |  | 125 | 125 |  |
| **Age, mean (SD), y** | 57.5 (15.8) | 61.2 (13.9) | 0.251 |  | 58.7 (15.5) | 59.2 (14.7) | 0.036 |
| **Male, no. (%)** | 84 (61.8) | 181 (69.6) | 0.166 |  | 77 (61.6) | 77 (61.6) | < 0.001 |
| **BMI,** **mean (SD), kg/m^2^** | 24.7 (4.5) | 24.0 (3.7) | 0.157 |  | 24.5 (4.5) | 24.1 (4.0) | 0.100 |
| **APACHE II score, mean (SD)** | 12.7 (4.9) | 15.7 (5.8) | 0.574 |  | 13.2 (4.7) | 13.0 (4.6) | 0.026 |
| **SOFA score, mean (SD)** | 6.3 (3.1) | 7.3 (3.2) | 0.318 |  | 6.5 (3.1) | 6.6 (3.2) | 0.028 |
| **GCS score, mean (SD)** | 9.9 (0.6) | 9.1 (1.9) | 0.553 |  | 9.9 (0.6) | 9.9 (0.7) | < 0.001 |
| **Comorbidities, no. (%)** | | | | | | | |
| Hypertension | 68 (50.0) | 155 (59.6) | 0.194 |  | 62 (49.6) | 61 (48.8) | 0.016 |
| Diabetes mellitus | 39 (28.7) | 78 (30.0) | 0.029 |  | 36 (28.8) | 34 (27.2) | 0.036 |
| Stroke | 14 (10.3) | 49 (18.8) | 0.244 |  | 14 (11.2) | 12 (9.6) | 0.052 |
| Coronary heart disease | 10 (7.4) | 27 (10.4) | 0.107 |  | 10 (8.0) | 12 (9.6) | 0.057 |
| Chronic kidney disease | 7 (5.1) | 31 (11.9) | 0.244 |  | 7 (5.6) | 7 (5.6) | < 0.001 |
| COPD | 7 (5.1) | 18 (6.9) | 0.075 |  | 7 (5.6) | 7 (5.6) | < 0.001 |
| **Primary diagnoses, no. (%)** | | | | | | | |
| Respiratory system | 44 (32.4) | 89 (34.2) | 0.040 |  | 42 (33.6) | 43 (34.4) | 0.017 |
| Circulatory system | 30 (22.1) | 41 (15.8) | 0.161 |  | 26 (20.8) | 27 (21.6) | 0.020 |
| Nervous system | 12 (8.8) | 63 (24.2) | 0.424 |  | 12 (9.6) | 12 (9.6) | < 0.001 |
| Digestive system | 22 (16.2) | 35 (13.5) | 0.076 |  | 20 (16.0) | 18 (14.4) | 0.045 |
| Others | 28 (20.6) | 32 (12.3) | 0.225 |  | 25 (20.0) | 25 (20.0) | < 0.001 |
| **Use of vasoactive drugs, no. (%)** | 27 (19.9) | 59 (22.7) | 0.069 |  | 27 (21.6) | 30 (24.0) | 0.057 |

**e-Table 5. Characteristics of patients with early versus late OBST before and after matching (Continued)**

| **Variables** | **Before matching** | | |  |  | **After matching** | | |
| --- | --- | --- | --- | --- | --- | --- | --- | --- |
|  | **Early OBST** | **Late OBST** | **SMD** |  |  | **Early OBST** | **Late OBST** | **SMD** |

**Abbreviations:** APACHE II = Acute Physiology and Chronic Health Evaluation II; BMI = body mass index; COPD = chronic obstructive pulmonary disease; CRRT = continuous renal replacement therapy; FSS-ICU = Functional Status Score for the Intensive Care Unit; GCS = Glasgow Coma Scale; ICU = intensive care unit; OBST = out-of-bed sitting training; SD = standard deviation; SMD = standardized mean difference; SOFA = Sequential Organ Failure Assessment.

| **Use of sedative drugs, no. (%)** | 38 (27.9) | 95 (36.5) | 0.185 |  | 37 (29.6) | 43 (34.4) | 0.103 |
| --- | --- | --- | --- | --- | --- | --- | --- |
| **Use of CRRT, no. (%)** | 13 (9.6) | 30 (11.5) | 0.064 |  | 12 (9.6) | 14 (11.2) | 0.052 |
| **Use of invasive ventilation, no. (%)** | 15 (11.0) | 101 (38.8) | 0.679 |  | 15 (12.0) | 18 (14.4) | 0.071 |
| **FSS-ICU score at** **OBST initiation, mean (SD)** | 15.0 (8.0) | 9.0 (8. 7) | 0.714 |  | 14.3 (7.8) | 13.8 (8.2) | 0.056 |
| **Study week of OBST initiation, mean (SD)** | 33.7 (17.0) | 32.2 (18.7) | 0.082 |  | 33.9 (16.9) | 32.8 (18.0) | 0.066 |

##

## e-Table 6. Types and incidence of potential safety events

| **Types** | **Before matching** | | |  | **After Matching** | | |
| --- | --- | --- | --- | --- | --- | --- | --- |
|  | **Total**  **2578 sessions** | **Early OBST**  **593 sessions** | **Late OBST**  **1985 sessions** |  | **Total**  **1359 sessions** | **Early OBST**  **553 sessions** | **Late OBST**  **806 sessions** |
| **Hemodynamic events, no. (%)** |  |  |  |  |  |  | |
| Heart rate variations >20% of baseline | 11 (0.43) | 2 (0.34) | 9 (0.45) |  | 8 (0.59) | 2 (0.36) | 6 (0.74) |
| Cardiac arrhythmias | 0 (0.00) | 0 (0.00) | 0 (0.00) |  | 0 (0.00) | 0 (0.00) | 0 (0.00) |
| Mean arterial pressures >140 mmHg | 0 (0.00) | 0 (0.00) | 0 (0.00) |  | 0 (0.00) | 0 (0.00) | 0 (0.00) |
| Mean arterial pressure <55 mmHg | 1 (0.04) | 1 (0.17) | 0 (0.00) |  | 0 (0.00) | 0 (0.00) | 0 (0.00) |
| **Respiratory events, no. (%)** |  |  |  |  |  |  |  |
| SpO_2_ <88% | 3 (0.12) | 0 (0.00) | 3 (0.15) |  | 2 (0.15) | 0 (0.00) | 2 (0.25) |
| Pneumothorax | 0 (0.00) | 0 (0.00) | 0 (0.00) |  | 0 (0.00) | 0 (0.00) | 0 (0.00) |
| **Neurological events, no. (%)^a^** | 0 (0.00) | 0 (0.00) | 0 (0.00) |  | 0 (0.00) | 0 (0.00) | 0 (0.00) |
| **Removal events, no. (%)** | 0 (0.00) | 0 (0.00) | 0 (0.00) |  | 0 (0.00) | 0 (0.00) | 0 (0.00) |
| **Other events, no. (%)** | 0 (0.00) | 0 (0.00) | 0 (0.00) |  | 0 (0.00) | 0 (0.00) | 0 (0.00) |
| **Total, no. (%)** | 15 (0.58) | 3 (0.51) | 12 (0.60) |  | 10 (0.74) | 2 (0.36) | 8 (0.99) |

**Abbreviation：**SpO_2_ = peripheral blood oxygen saturation.

^a^ One patient who underwent early OBST experienced a transient loss of consciousness. Based on clinical evaluation and underlying pathology, this event was determined to be unrelated to the OBST session and attributed to natural disease progression.

## e-Table 7. Primary outcome and secondary outcomes before propensity score matching

| **Variables** | **Difference  (reference: Late OBST)** | **Standard Error** | **95% CI** | ***P* value** |
| --- | --- | --- | --- | --- |
| **Primary outcome** |  |  |  |  |
| Potential safety events rate, % | RD: −0.099 | 0.003 | −0.764 to 0.566 | **<0.001** |
| **Secondary outcomes** |  |  |  |  |
| Change in FSS-ICU score | MD: 1.990 | 0.518 | 0.973 to 3.008 | **<0.001** |
| Change in arterial blood gas |  |  |  |  |
| PaO_2,_ mmHg | MD: −3.482 | 3.332 | −10.034 to 3.070 | 0.297 |
| PaCO_2,_ mmHg | MD: −0.259 | 0.750 | −1.734 to 1.216 | 0.730 |
| PaO_2_/FiO_2_, mmHg | MD: −5.404 | 9.341 | −23.767 to 12.960 | 0.563 |
| Lac, mmol/L | MD: 0.030 | 0.096 | −0.160 to 0.219 | 0.759 |
| Duration per OBST session, min | MD: 10.221 | 1.770 | 6.751 to 13.691 | **< 0.001** |
| Number of OBST sessions | MD: −3.274 | 0.594 | −4.441 to −2.107 | **< 0.001** |
| ICU length of stay, d | MD: −5.299 | 0.793 | −6.857 to −3.740 | **< 0.001** |
| Ventilator-free days | MD: 6.251 | 2.636 | 1.029 to 11.472 | **0.019** |
| Weaning success, % | RD: 0.092 | 0.074 | −0.053 to 0.237 | 0.694 |
| ICU mortality, % | RD: −0.031 | 0.011 | −0.052 to −0.010 | 0.055 |
| 28−day post-ICU mortality, % ^a^ | RD: −0.050 | 0.019 | −0.088 to −0.013 | **0.040** |

**Abbreviations:** FiO_2_ = fraction of inspired oxygen; FSS-ICU = Functional Status Score for the Intensive Care Unit; ICU = intensive care unit; IQR = interquartile range; Lac = lactate; MD = mean difference; OBST = out-of-bed sitting training; PaCO_2_ = arterial carbon dioxide partial pressure; PaO_2_ = arterial oxygen partial pressure; RD = risk difference.

^a^ Before matching, 28-day post-ICU mortality data were missing for 4 patients with early OBST and 8 with late OBST.

## e-Table 8. Sensitivity analysis of outcomes by study week in the propensity score–matched cohort

| **Variables** | **Difference  (reference: Late OBST)** | **Standard Error** | **95% CI** | ***P* value** |
| --- | --- | --- | --- | --- |
| **Primary outcome** |  |  |  |  |
| Potential safety events rate, % | RD: −0.783 | 0.004 | −1.654 to 0.087 | **<0.001** |
| **Secondary outcomes** |  |  |  |  |
| Change in FSS-ICU score | MD: 1.332 | 0.559 | 0.230 to 2.433 | **0.018** |
| Change in arterial blood gas |  |  |  |  |
| PaO_2,_ mmHg | MD: −2.259 | 3.834 | −9.809 to 5.291 | 0.556 |
| PaCO_2,_ mmHg | MD: 0.506 | 0.839 | −1.154 to 2.165 | 0.548 |
| PaO_2_/FiO_2_, mmHg | MD: −7.094 | 11.045 | −28.846 to 14.658 | 0.521 |
| Lac, mmol/L | MD: 0.010 | 0.115 | −0.217 to 0.237 | 0.931 |
| Duration per OBST session, min | MD: 3.753 | 2.337 | −0.832 to 8.337 | 0.109 |
| Number of OBST sessions | MD: −2.015 | 0.556 | −3.109 to −0.921 | **< 0.001** |
| ICU length of stay, d | MD: −2.720 | 0.688 | −4.081 to −1.358 | **< 0.001** |
| Ventilator-free days | MD: 2.516 | 5.026 | -8.492 to 13.524 | 0.626 |

**Abbreviations:** FiO_2_ = fraction of inspired oxygen; FSS-ICU = Functional Status Score for the Intensive Care Unit; ICU = intensive care unit; IQR = interquartile range; Lac = lactate; MD = mean difference; OBST = out-of-bed sitting training; PaCO_2_ = arterial carbon dioxide partial pressure; PaO_2_ = arterial oxygen partial pressure; RD = risk difference.

## e-Table 9. Secondary outcomes

| **Variables** | **Before matching** | |  | **After matching** | |
| --- | --- | --- | --- | --- | --- |
|  | **Early OBST**  **N = 136** | **Late OBST**  **N = 260** |  | **Early OBST**  **N = 125** | **Late OBST**  **N = 125** |
| **Change in FSS-ICU score, median (IQR)** | 6.0 (3.0 to 8.0) | 4.0 (0.0 to 6.0) |  | 6.0 (3.0 to 9.0) | 4.0 (2.0 to 8.0) |
| **Change in arterial blood gas, median (IQR)** | | | |  |  |
| PaO_2,_ mmHg | 4.7 (−10.5 to 21.7) | 7.8 (−9.2 to 26.9) |  | 4.6 (−10.4 to 18.8) | 2.6 (−11.6 to 23.3) |
| PaCO_2,_ mmHg | −0.8 (−4.0 to 3.0) | −0.7 (−5.6 to 3.5) |  | −0.7 (−3.8 to 3.1) | −1.1 (−6.1 to 3.4) |
| PaO_2_/FiO_2_, mmHg | 47.9 (2.8 to 100.8) | 49.6 (1.9 to 115.9) |  | 46.0 (1.8 to 89.4) | 52.4 (1.6 to 123.0) |
| Lac, mmol/L | 0.0 (−0.5 to 0.4) | −0.1 (−0.6 to 0.4) |  | 0.0 (−0.4 to 0.4) | 0.0 (−0.6 to 0.4) |
| **Duration per OBST session, median (IQR), min** | 65.0 (47.0 to 94.0) | 60.0 (45.0 to 80.0) |  | 67.0 (48.0 to 94.0) | 60.5 (49.0 to 90.0) |
| **Number of OBST sessions, median (IQR)** | 4.0 (2.0 to 5.2) | 6.0 (3.0 to 9.0) |  | 4.0 (2.0 to 5.0) | 5.0 (3.0 to 8.0) |
| **ICU length of stay, median (IQR), d** | 4.0 (2.0 to 6.0) | 7.0 (4.0 to 14.0) |  | 4.0 (2.0 to 6.0) | 6.0 (3.0 to 9.0) |
| **Ventilator-free days, median (IQR)** | 23.0 (21.0 to 25.0) | 19.0 (6.0 to 24.0) |  | 23.0 (21.0 to 25.0) | 19.5 (14.0 to 21.8) |
| **Weaning success, no. (%)** | 14 (93.3) | 85 (84.2) |  | 14 (93.3) | 17 (94.4) |
| **ICU mortality, no. (%)** | 0 (0.0) | 8 (3.1) |  | 0(0.0) | 2 (1.6) |
| **28-day post-ICU mortality, no. (%)^a^** | 2 (1.5) | 16 (6.6) |  | 2 (1.7) | 6 (5.1) |

**Abbreviations**：FiO_2_ = fraction of inspired oxygen; FSS-ICU = Functional Status Score for the Intensive Care Unit; ICU = intensive care unit; IQR = interquartile range; Lac = lactate; OBST = out-of-bed sitting training; PaCO_2_ = arterial carbon dioxide partial pressure; PaO_2_ = arterial oxygen partial pressure.

^a^ Before matching, 28-day post-ICU mortality data were missing for 4 patients with early OBST and 8 with late OBST; after matching, missingness was observed for 4 and 5 patients, respectively.


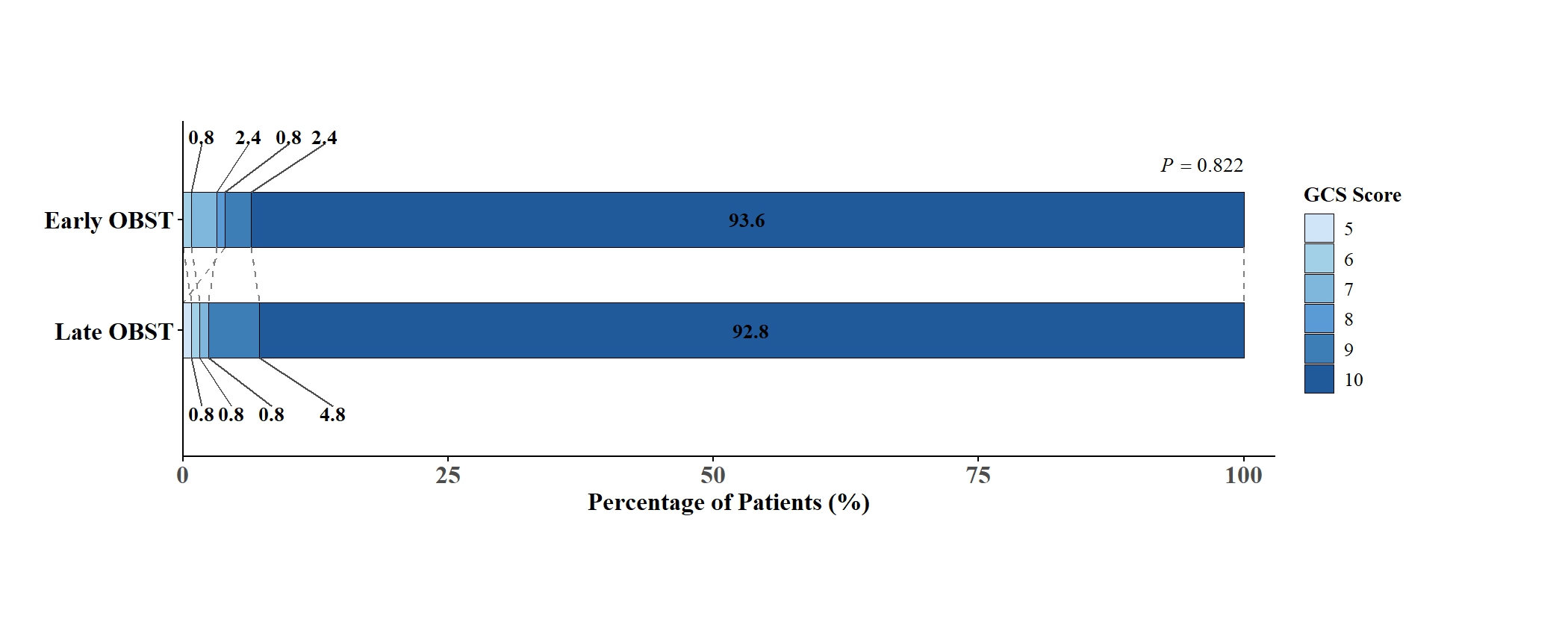


## e-Figure 1. GCS score distribution according to OBST timing

**Abbreviation:** GCS = Glasgow Coma Scale.


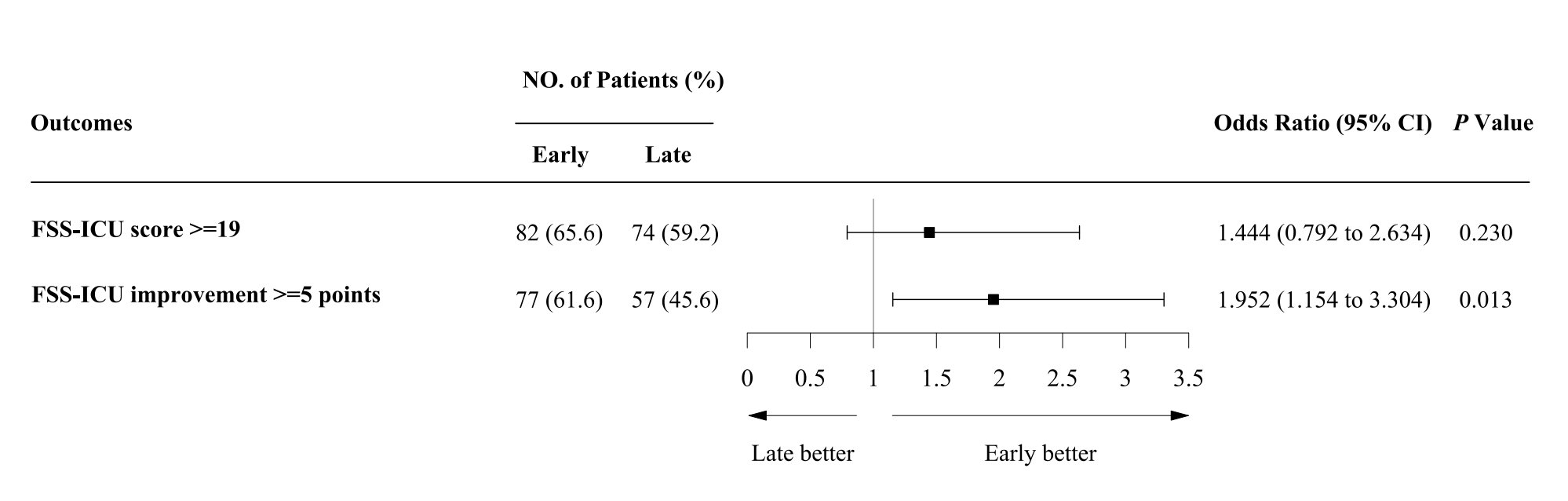


## e-Figure 2. Odds ratios for functional outcomes according to OBST timing

**Abbreviations:** FSS-ICU = Functional Status Score for the Intensive Care Unit; OBST = out-of-bed sitting training


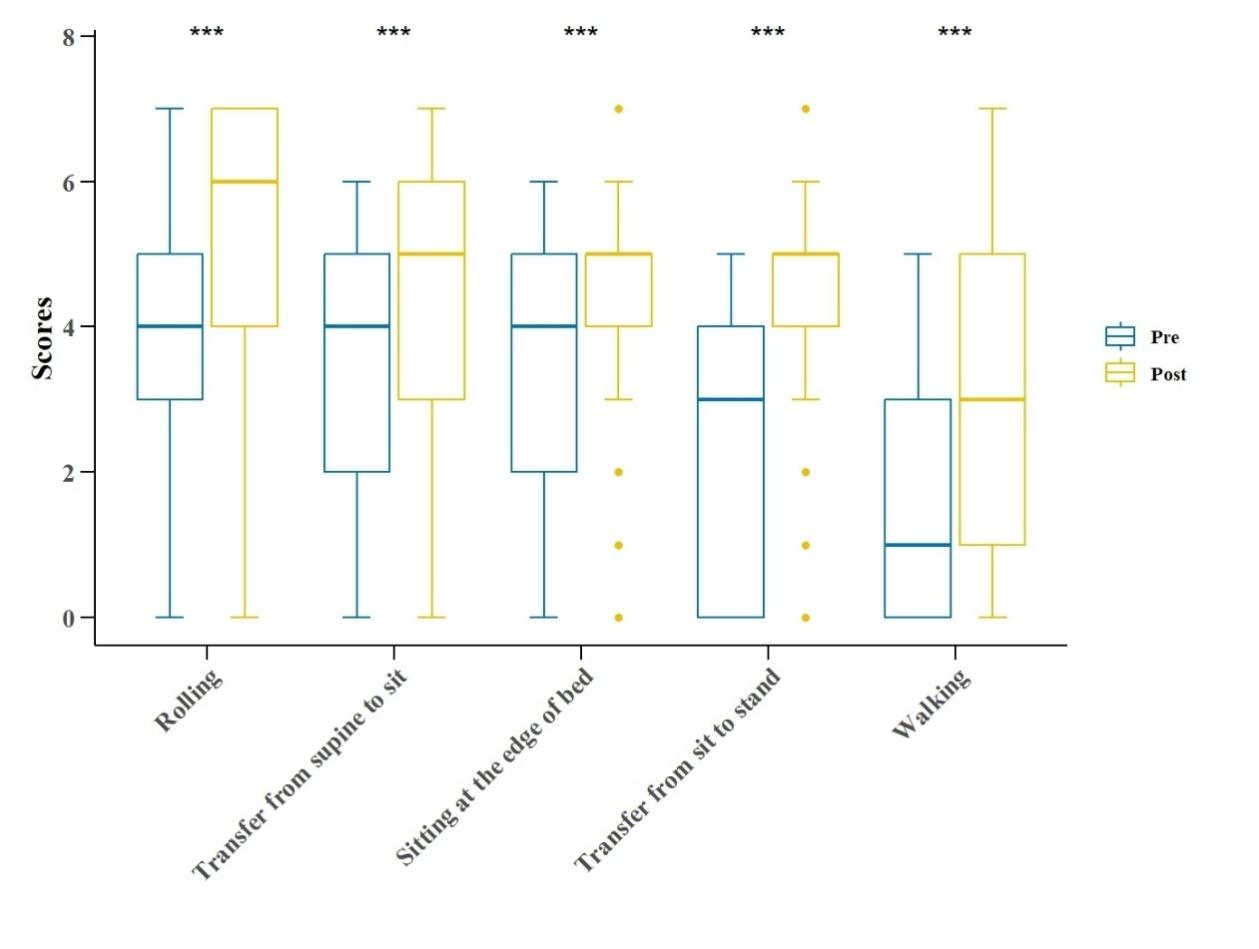


## e-Figure 3. Changes in FSS-ICU domain scores among patients with early OBST

Box plots represent median functional status scores, with interquartile range (IQR) indicated by the box and whiskers extending to 1.5× IQR; outliers are marked as individual points beyond the whiskers. ***P < 0.001 (Wilcoxon signed-rank test).

**Abbreviation:** FSS-ICU = Functional Status Score for the Intensive Care Unit.


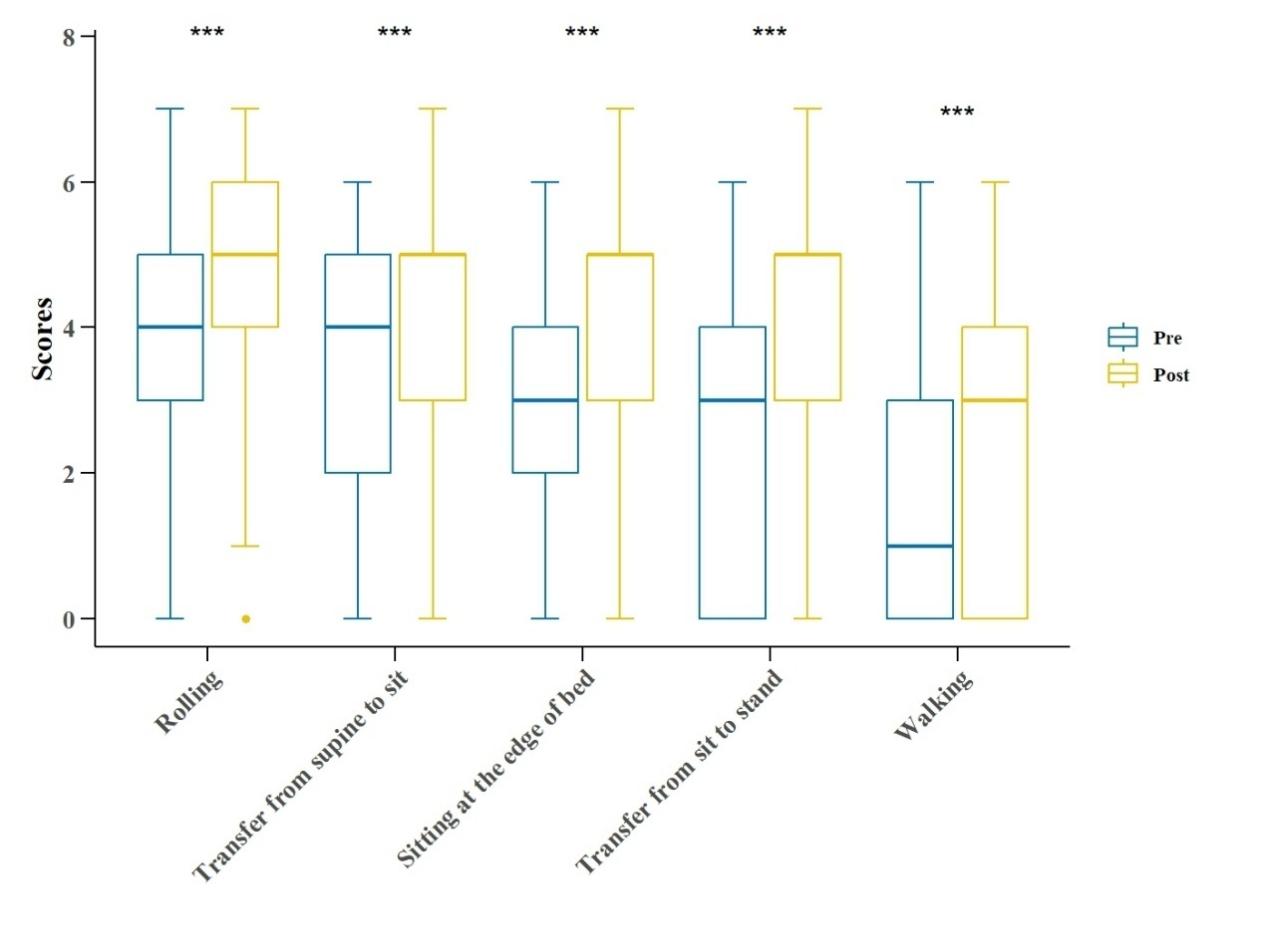


## e-Figure 4. Changes in FSS-ICU domain scores among patients with late OBST

Box plots represent median functional status scores, with interquartile range (IQR) indicated by the box and whiskers extending to 1.5× IQR; outliers are marked as individual points beyond the whiskers. ***P < 0.001 (Wilcoxon signed-rank test).

**Abbreviation:** FSS-ICU = Functional Status Score for the Intensive Care Unit.


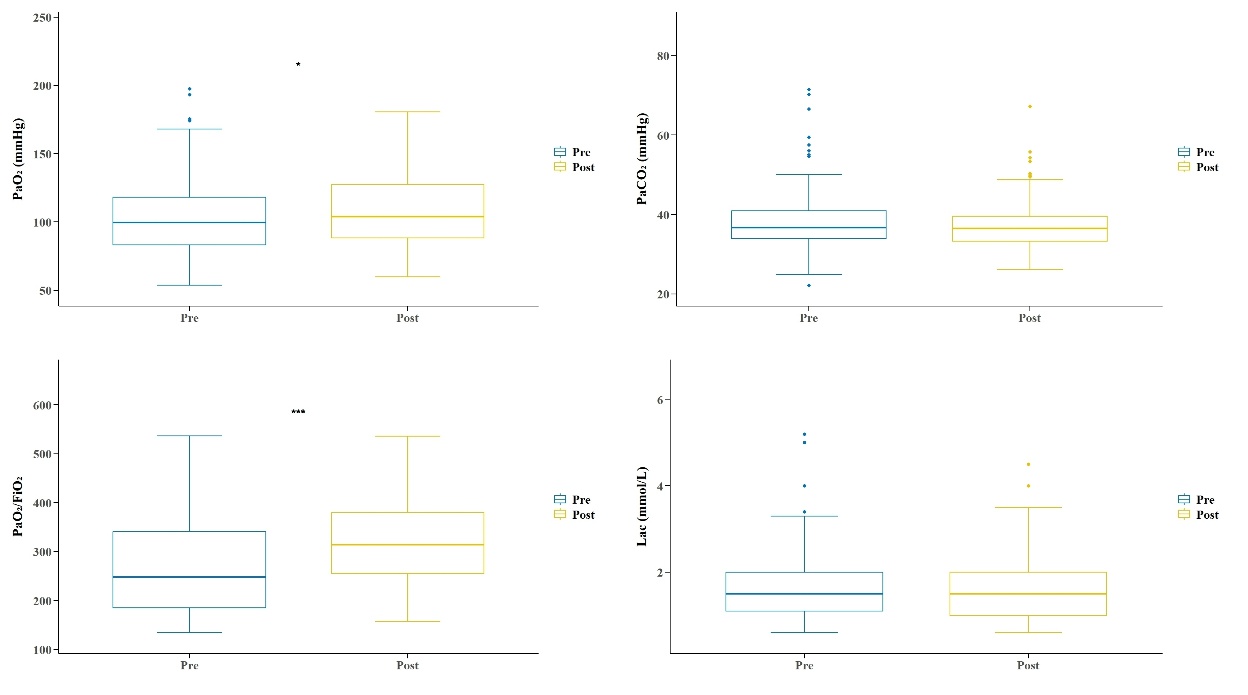


## e-Figure 5. Changes in arterial blood gas parameters among patients with early OBST

Box plots represent median arterial blood gas parameters, with interquartile range (IQR) indicated by the box and whiskers extending to 1.5× IQR; outliers are marked as individual points beyond the whiskers. *P < 0.05 and ***P < 0.001 (Wilcoxon signed-rank test).

**Abbreviations:** FiO_2_ = fraction of inspired oxygen; PaCO_2_ = arterial carbon dioxide partial pressure; PaO_2_ = arterial oxygen partial pressure; Lac = lactate.


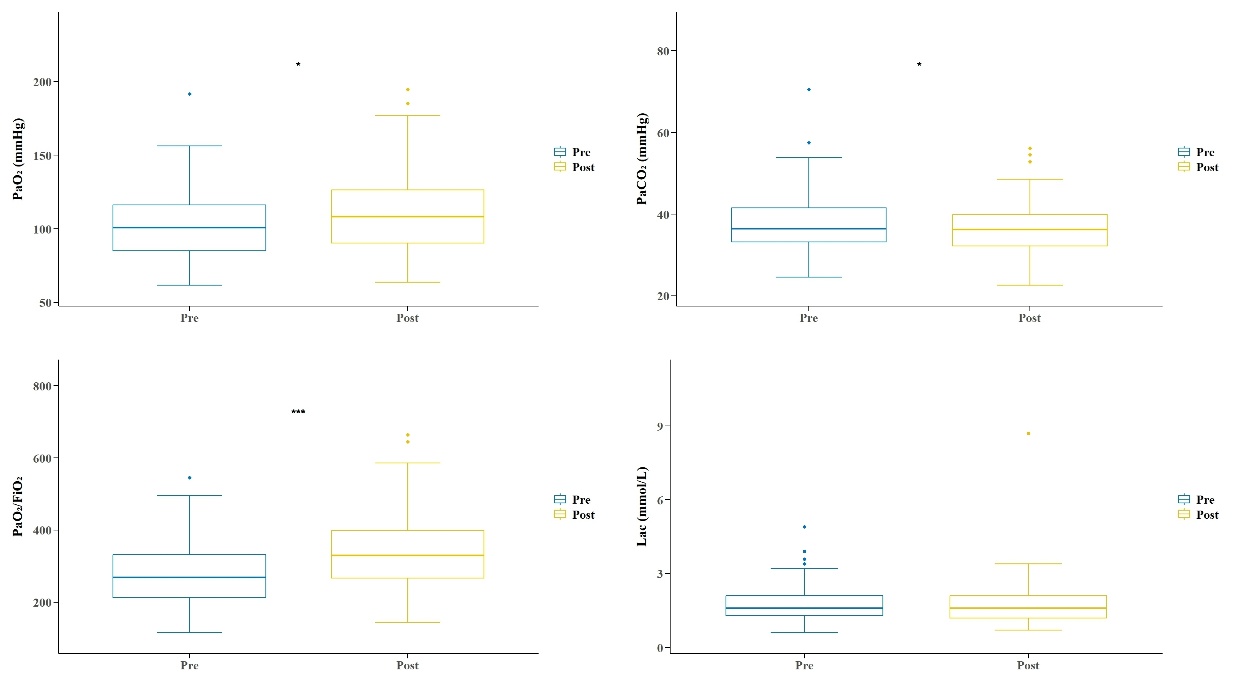


## e-Figure 6. Changes in arterial blood gas parameters among patients with late OBST

Box plots represent median arterial blood gas parameters, with interquartile range (IQR) indicated by the box and whiskers extending to 1.5× IQR; outliers are marked as individual points beyond the whiskers. *P < 0.05 and ***P < 0.001 (Wilcoxon signed-rank test).

**Abbreviations:** FiO_2_ = fraction of inspired oxygen; PaCO_2_ = arterial carbon dioxide partial pressure; PaO_2_ = arterial oxygen partial pressure; Lac = lactate.

## References:

1. Hodgson CL, Stiller K, Needham DM, Tipping CJ, Harrold M, Baldwin CE, et al. Expert consensus and recommendations on safety criteria for active mobilization of mechanically ventilated critically ill adults. Crit Care. 2014;18:658.

2. Unoki T, Hayashida K, Kawai Y, Taito S, Ando M, Iida Y, et al. Japanese Clinical Practice Guidelines for Rehabilitation in Critically Ill Patients 2023 (J-ReCIP 2023). J Intensive Care. 2023;11:47.
